# Supplementary material for: High-resolution mass measurements of single budding yeast reveal linear growth segments
Source: Nat Commun. 2022 Jun 22;13:3483. doi: 10.1038/s41467-022-30781-y (PMC9217925; doi:10.1038/s41467-022-30781-y)
Supplement: Supplementary file 1 — Supplementary Information [file 41467_2022_30781_MOESM1_ESM.pdf]

Supplementary Information

for

High-resolution mass measurements of single budding yeast reveal linear growth segments

Andreas P. Cuny[1,2,$], K. Tanuj Sapra[1,7,$], David Martinez-Martin[1,3,4,$,*], Gotthold Fläschner[1,$], Jonathan D. Adams[1], Sascha Martin[5], Christoph Gerber[6], Fabian Rudolf[1,2]* and Daniel J. Müller[1]*

[1]Eidgenössische Technische Hochschule (ETH) Zürich, Department of Biosystems Science and Engineering, 4058 Basel, Switzerland.
[2]Swiss Institute of Bioinformatics (SIB), 4058 Basel, Switzerland.
[3]The University of Sydney, School of Biomedical Engineering, NSW 2006 Sydney, Australia.
[4]The University of Sydney Nano Institute (Sydney Nano), The University of Sydney, Sydney, Australia
[5]University of Basel, Department of Physics, 4056 Basel, Switzerland.
[6]University of Basel, Swiss Nanoscience Institute (SNI), 4056 Basel, Switzerland.
[7]Present address: Quantum-Si Inc., 530 Old Whitfield Street, Guilford, CT 06437, USA
[$]Authors contributed equally
*Correspondence: (D.M.-M.) david.martinezmartin@sydney.edu.au; (F.R.) fabianrudolf@icloud.com; (D.J.M.) daniel.mueller@bsse.ethz.ch

Supplementary Figures

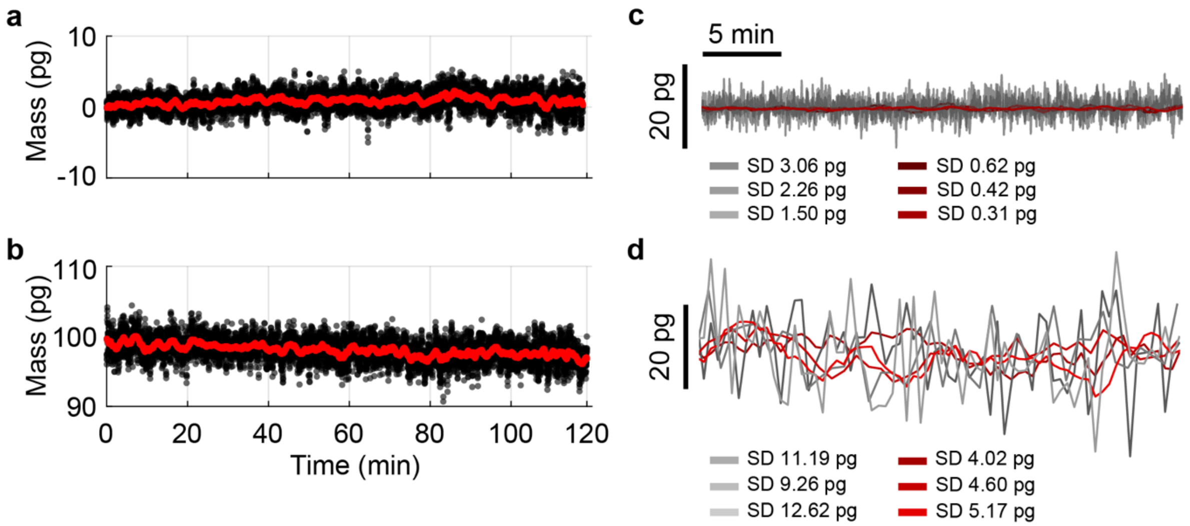

Figure 1. Mass measurements using the picobalance show long-term stability and approaches femtogram mass resolution. a, Mass measurement of a microcantilever (no cell attached) recorded in cell culture medium (Methods) for 120 min at a high time resolution of 10 ms in the continuous mode. **b**, Mass measurement of a non-growing yeast cell attached to the cantilever in minimal synthetic defined medium (SDmin) in the continuous mode. **a**, **b**, Shown are raw (black dots) and smoothened (100 s moving window, red line) data. **c**, Three independent mass measurements (n = 3) recorded using the continuous mode, show resolution of 1.5 pg, 3.1 pg and 2.3 pg as determined by their standard deviations (SD) of the noise. Smoothing (100 s moving window) increases the resolution (SD) to 0.3 pg, 0.6 pg and 0.4 pg. **d**, Three independent mass measurements (n = 3) recorded using the sweep mode show a resolution (SD) of 11.2, 9.3 and 12.6 pg. Smoothing (350 s moving window) increases the resolution (SD) to 4.0 pg, 4.6 pg and 5.2 pg. **c**, **d**, Raw data (grey lines), smoothed (red lines); black scale bars for the mass (20 pg, vertical) and time (5 min, horizontal).

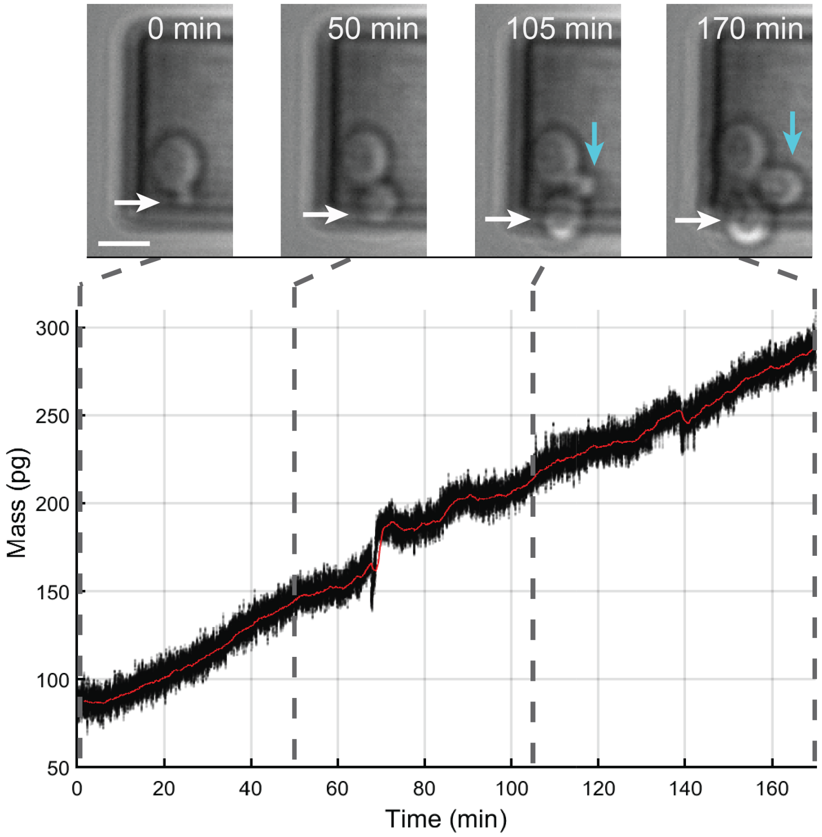

Figure 2. **Yeast cells growing over multiple cell cycles (generations) on the microcantilever during mass measurements recorded using the high-resolution continuous mode.** Mass and morphology of a budding yeast cell over the time course of more than 2 h. The cell attached to the microcantilever initiates several daughter cells, which is monitored by both mass and DIC microscopy for the time course of ≈ 170 min. Generation of the first daughter cell is indicated with a white arrow and of the second daughter cell is indicated with a blue arrow. The black trace is the raw data showing the total mass of the growing cell(s) acquired every 10 ms at a mass resolution of ≈ 4 pg, while the red trace shows the smoothened raw data (100 s moving window) approaching a mass resolution of ≤ 0.5 pg. The *S. cerevisiae* cell had been attached to the cantilever of the picobalance and the mass was recorded using the continuous mode in yeast culture medium under cell culture conditions as described (Methods). See also Supplementary Movie 2. Scale bar (white line), 5 μm.

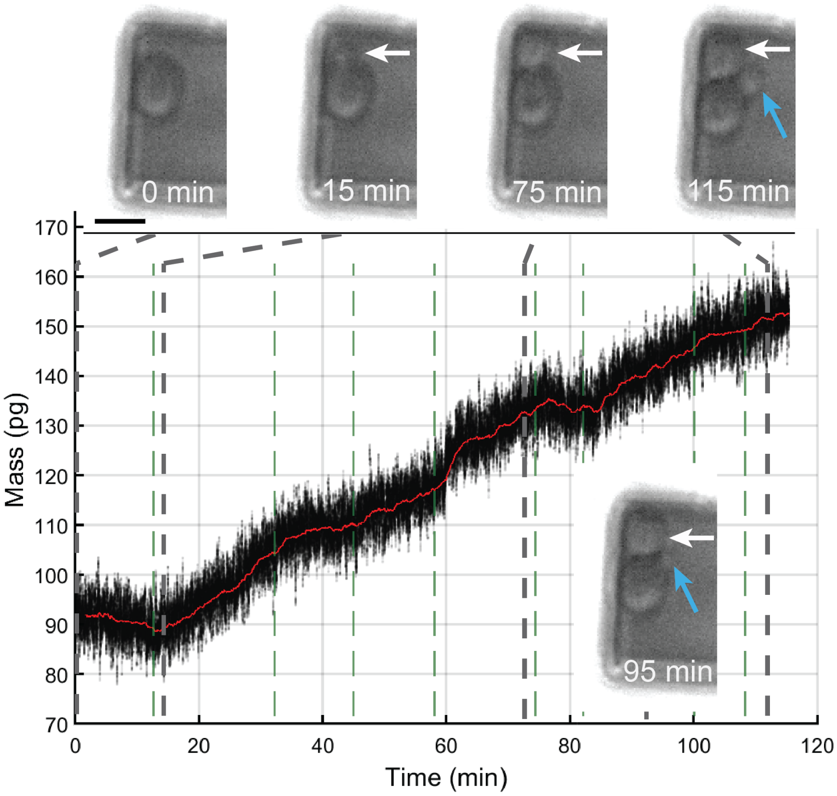

Figure 3. ***S. cerevisiae* increases mass in segments at different rates over the cell cycle for consecutive budding events.** Segmented growth is not limited to budding (S/G2/M) phase and can be observed across the cell cycle. Monitoring the mass and morphology of a single *S. cerevisiae* cell for two hours. While measuring the total mass, differential interference contrast (DIC) images observe the budding process of the cell attached to the cantilever. The first budding process is indicated with a white arrow and the second bud is indicated with a blue arrow. The black raw data shows the mass of the cellular process acquired every 10 ms at a mass resolution of ≈ 3 pg, while the red curve shows the smoothened raw data (100 s moving window) approaching a mass resolution of ≤ 0.5 pg. Segments (vertical green dashed line). Before budding, between 0 min to ≈ 15 min, the yeast cell keeps its total mass roughly constant. With the emergence of the bud at ≈ 15 min, the yeast cell starts increasing the mass roughly linear. After ≈ 85 min, the budding of a second daughter cell is observed. The yeast cell had been attached to the cantilever of the picobalance and the measurement was recorded using the continuous mode in yeast culture medium as described (Methods). Scale bar (black line), 5 µm. See also Supplementary Movie 3.

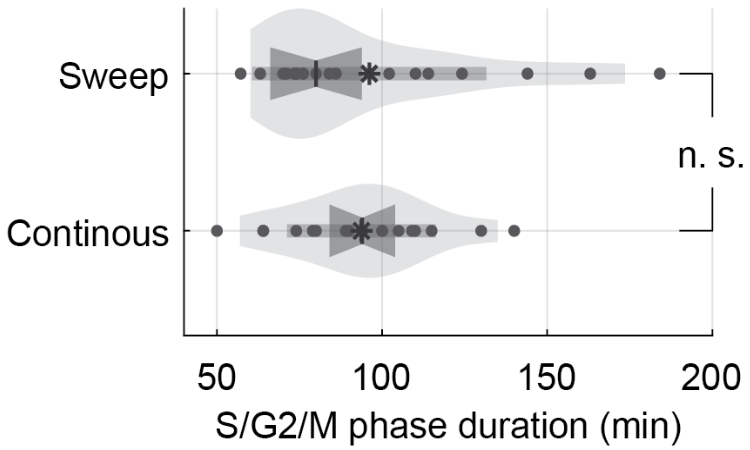

Figure 4. High-resolution continuous mode and sweep mode mass measurements do not impair growth of *S. cerevisiae* cells during S/G2/M phase. The S/G2/M (bud growth) phases of individual cells have been determined by optical microscopy for both sweep mode (n = 19 cells) and continuous mode (n = 19 cells) mass measurements. For the sweep mode we used the fluorescently labeled cell cycle marker Myo1 to determine the bud growth phase whereas for the continuous mode we used the DIC images (The phases have been determined by three authors independently). Yeast cells were exposed to the blue laser exciting the microcantilever (continuous mode) and on intervals of 30–50 s duration (sweep mode). The duration of the S/G2/M phase of the yeast cells characterized in the higher resolution continuous mode (mean = 93.7, SD = 22.5) is not significantly different (n.s., two sided t-test $t(36) = -0.2365$, $p = 0.8144$, $d = -0.077$) as well as a Kolmogorov-Smirnov test) from the duration observed in the sweep mode (mean = 96.0, SD = 35.4) where cells are exposed to less light during their cell cycle with alpha = 0.05. Raw data for sweep and continuous mode (black dots) with mean (96.1 min, 93.8 min, black star) and median (80.0 min, 94.0 min, vertical black line) with 95% confidence interval ([66.2; 93.8], [84.1; 104.0] dark grey edges) and kernel density (light grey, from the 1st percentile $q(0.01)$ to the 99th percentile $q(0.99)$).

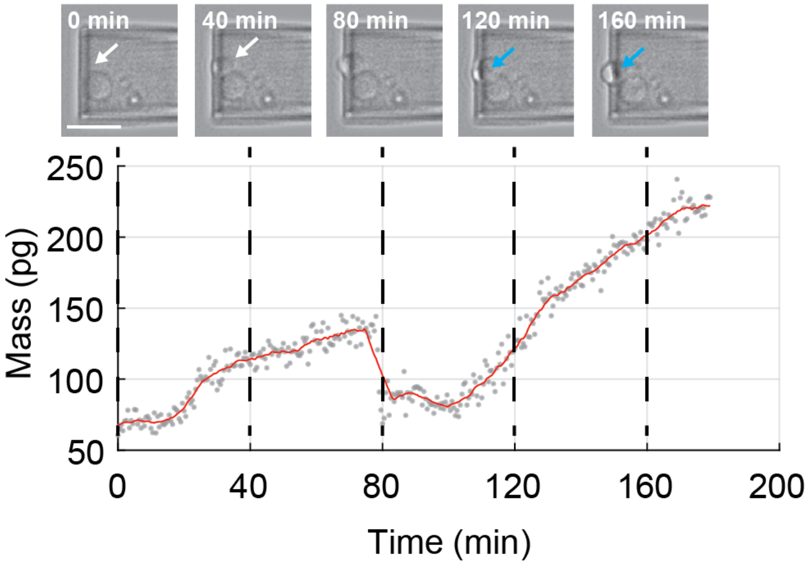

Figure 5. Mass and morphology of several budding yeast cell generations recorded using the sweep mode. The first-generation of a budding daughter cell by a mother cell is indicated with white arrows on the DIC images, while the blue arrows indicate a second-generation daughter cell. At ≈ 80 min, the bud loses attachment to the cantilever after cell division and the mass drops to that of the mother cell in the first G1 phase. The loss of the daughter cell is followed by a new budding event (blue arrows). The continuous budding process confirms the non-invasive character of the experimental conditions. The total mass, monitored by directly sweeping the cantilever phase, shows lower time and mass resolution compared to the total mass monitored in the continuous mode and confirms that the mass detected using the continuous mode is free of phase instabilities. Raw data (grey dots) measured every 30 s, moving average (60 s moving window, red line). See also Supplementary Movie 5. Scale bar (white line), 10 μm.

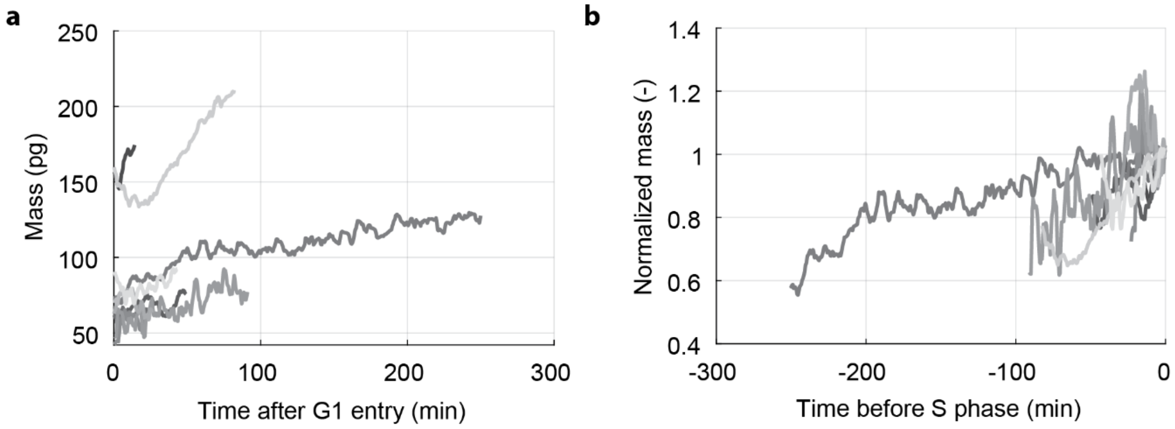

Figure 6. Mass curves recorded of single *S. cerevisiae* cells in the G1 phase. a, Mass curves for single yeast cells progressing through the G1 phase acquired using the sweep mode from n = 10 independent experiments. The time 0 min corresponds to the start of the G1 phase (Whi5 fluorescent signal in nucleus). The cell cycle phase was determined by fluorescence. **b**, Mass curves in **a** normalized to their respective end mass. The time 0 min corresponds to the beginning of S phase.

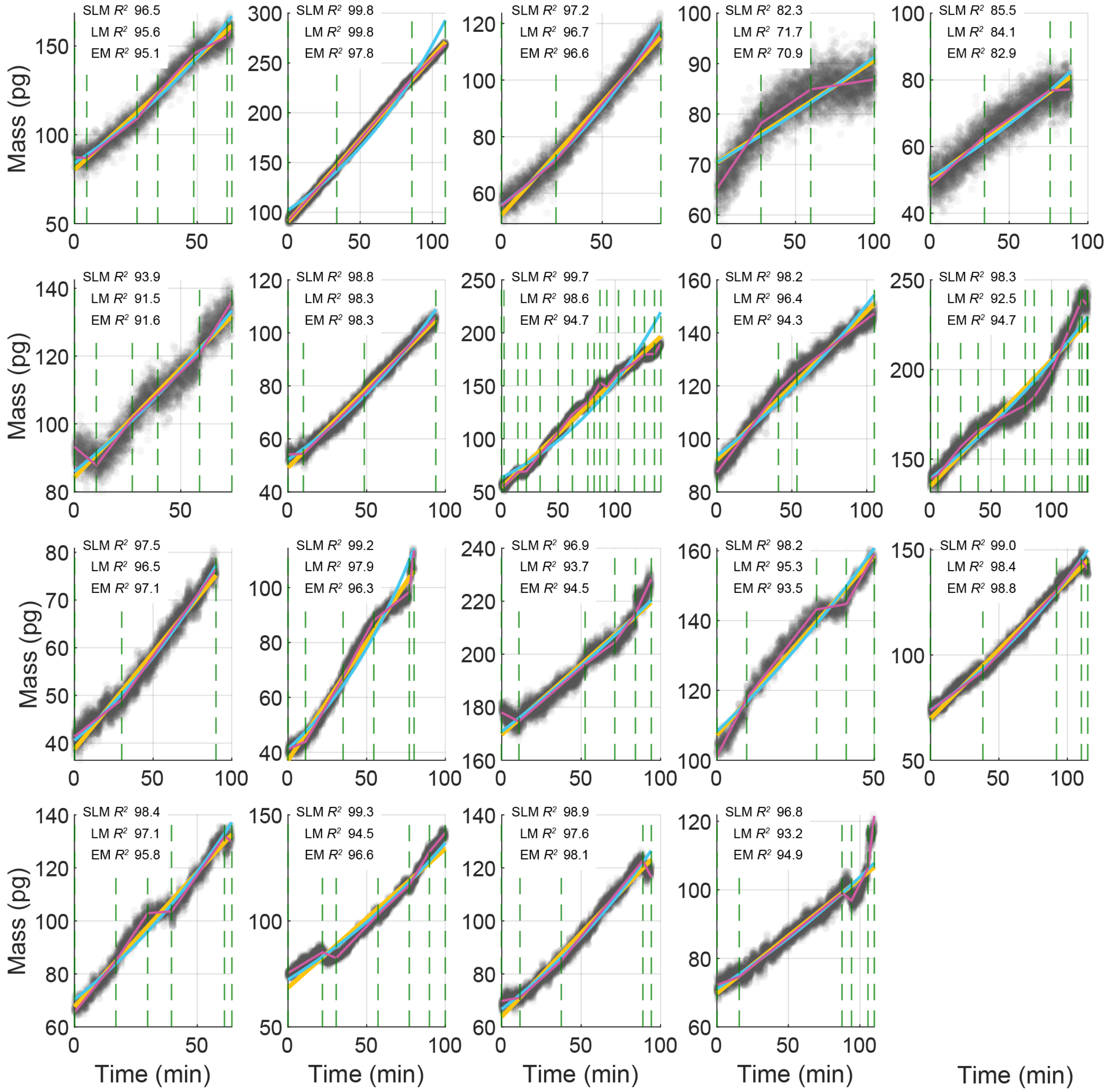

Figure 7. **Comparison of different regressions fitting the growth data recorded of single yeast cells progressing through the S/G2/M phase using the continuous mode.** Mass *versus* time curves of $n = 19$ *S. cerevisiae* cells recorded using the high-resolution continuous mode (10 ms) from independent experiments. Shown are raw mass *versus* time data (dark grey dots), linear segments fitted by the SLM model (violet line), and its segments (vertical dashed lines, green), linear model (LM, yellow line) and the exponential model (EM, cyan line). Due to minimal differences between LM and EM, the fitted lines are hard to distinguish visually. The coefficient of determination R^2 is given for each model. For all measured yeasts, the SLM model provides the highest R^2.

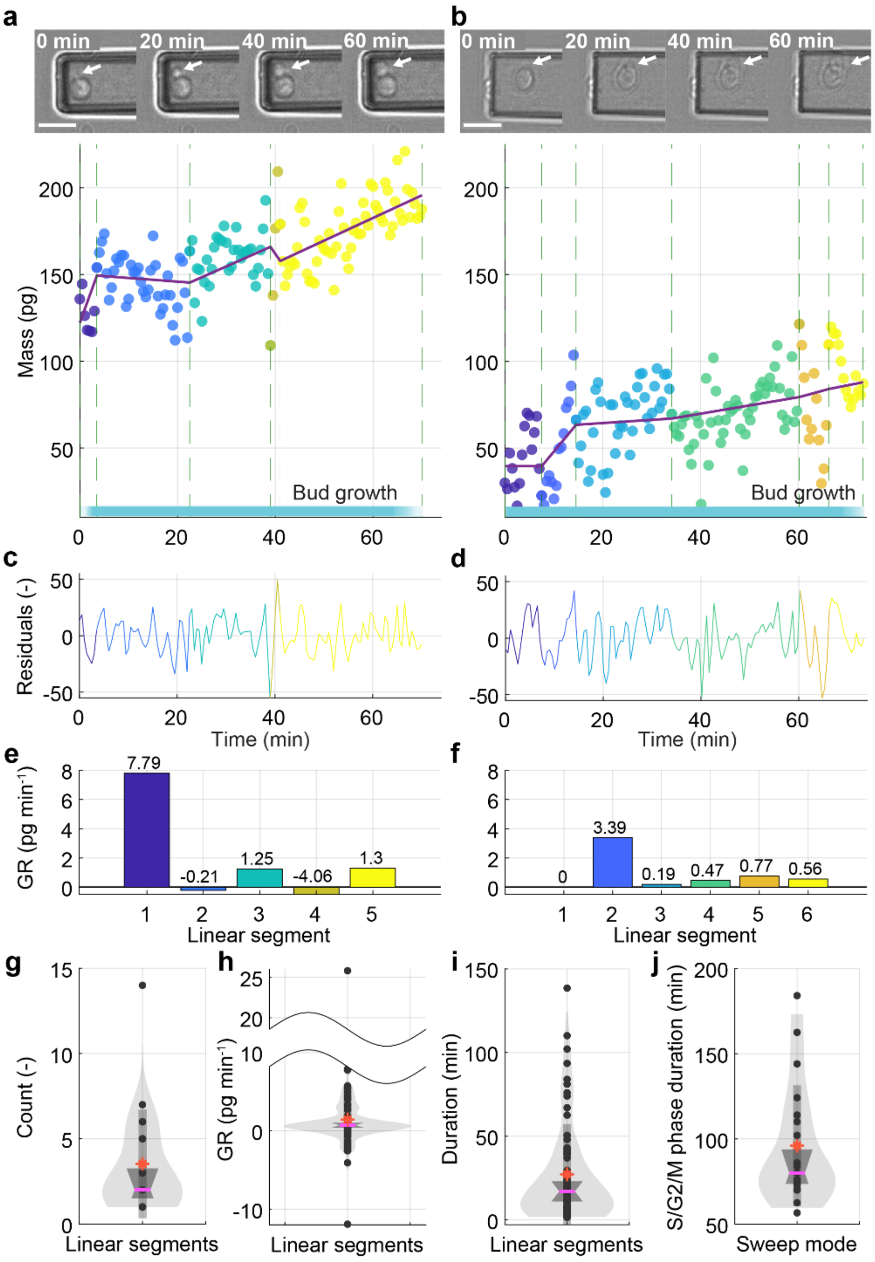

Figure 8. Budding single *S. cerevisiae* cells increase mass in linear segments. a, **b**, Mass *versus* time curves (growth curves) of two *S. cerevisiae* cells progressing through the S/G2/M phase recorded using the sweep mode. The growth curves are segmented (green vertical dashed lines) into segments of linear growth (colored) by a segmented linear regression model (SLM) (violet line). Dots are raw data recorded every ≈ 30 s using the sweep mode. Scale bars (white), 10 µm. Cyan bars indicate where cells are in the S/G2/M phase when bud growth is observed. **c**, **d**, Residuals of fitting the SLM to the raw data of the growth curves. **e**, **f**, Growth rate (GR) of linear growth segment (colored) shown in **a**, **b**. GR values given above the bars. **g-j**, Growth analysis of mass measurements of (n = 19) biologically independent yeast cells progressing through the S/G2/M phase recorded using the sweep mode in (n = 19) independent experiments. Data represented with violin plots show the raw data (grey dots), mean (orange star), median (pink line), mean and standard deviation (mean ± SD, medium grey vertical box), distribution as kernel density (light grey

area from the 1st percentile $q(0.01)$ to the 99th percentile $q(0.99)$) and the 95% confidence interval (CI, dark grey edges of hourglass hexagon). **g**, Number of linear segments of growth curves detected in all 19 cells. 4.0 (mean, orange star) and 2.0 (median, pink horizontal line) linear segments are observed per cell with 95% CI=[0.7, 3.3] (dark grey edges of hourglass hexagon). The kernel density (light grey area) and the standard deviation (SD, medium grey vertical box) show the distribution of the data. **h**, Growth rate (GR) distribution (slopes of linear segments) found in the growth curves of all cells. Median is 0.7 pg min^{-1} with 95% CI=[0.4, 1.0]. **i**, Duration (time span) of the linear segments of the growth curves of all cells. Median is 17.0 min with 95% CI=[11.0, 23.0]. **j**, S/G2/M phase durations for all 19 cells. Median is 80.0 min with 95% CI=[66.2, 93.8]. Black circles in **g-j** represent measurements of single yeast cells.

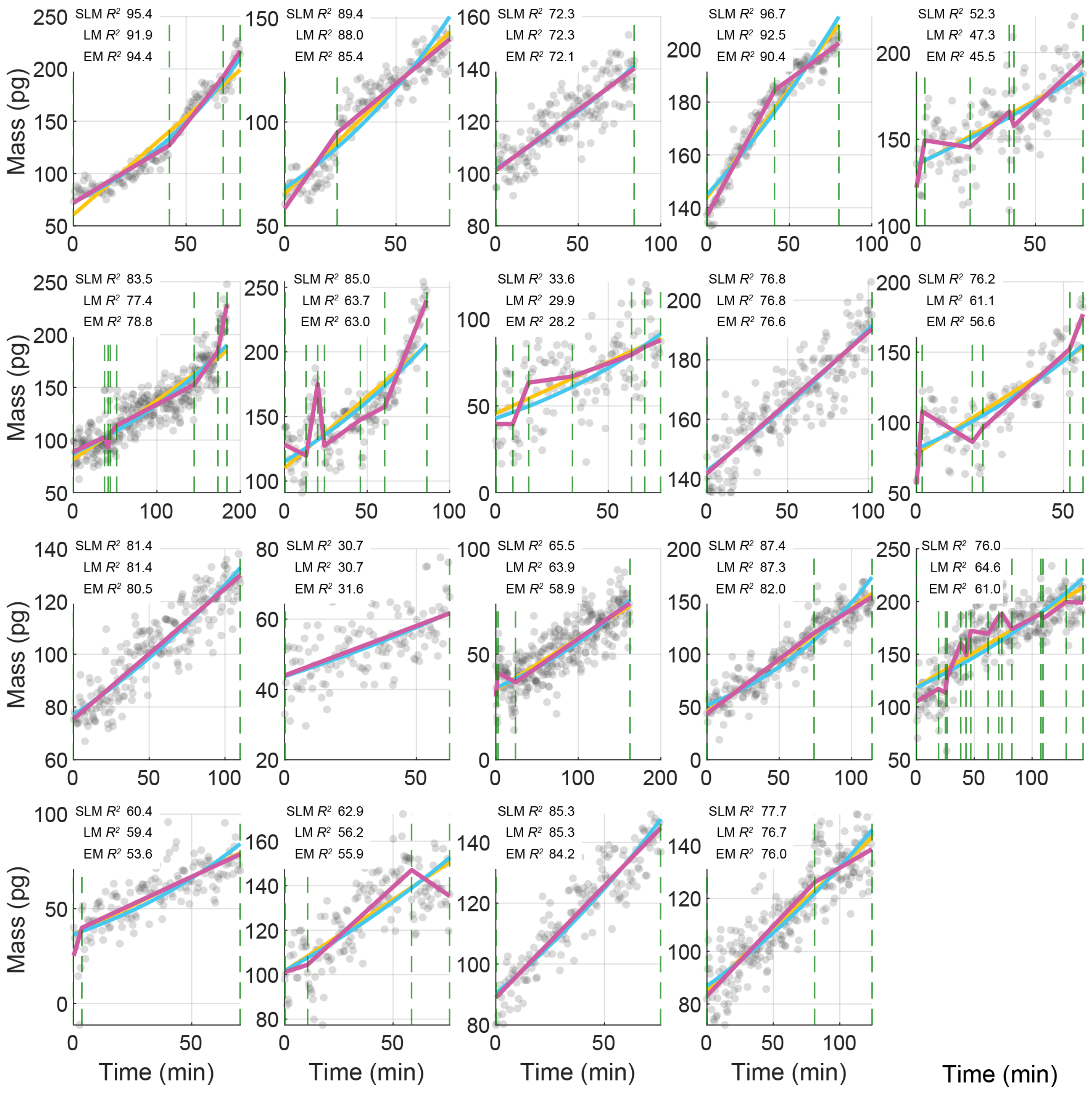

Figure 9. **Comparison of different regressions fitting the growth data recorded of single yeast cells progressing through the S/G2/M phase using the sweep mode.** Mass *versus* time curves of 19 *S. cerevisiae* cells recorded using the sweep mode. Shown are raw mass *versus* time data (dark grey dots), linear segments fitted by the SLM model (violet line), and its segments (vertical dashed lines, green), linear model (yellow line, LM) and the exponential model (cyan line, EM). Due to minimal differences between LM and EM, the fitted lines can be hard to distinguish. The coefficient of determination R^2 is given for each model. As in Supplementary Fig. 7, the SLM model has the highest R^2 value, however, due to the lower resolution of the raw data the R^2 are generally lower.

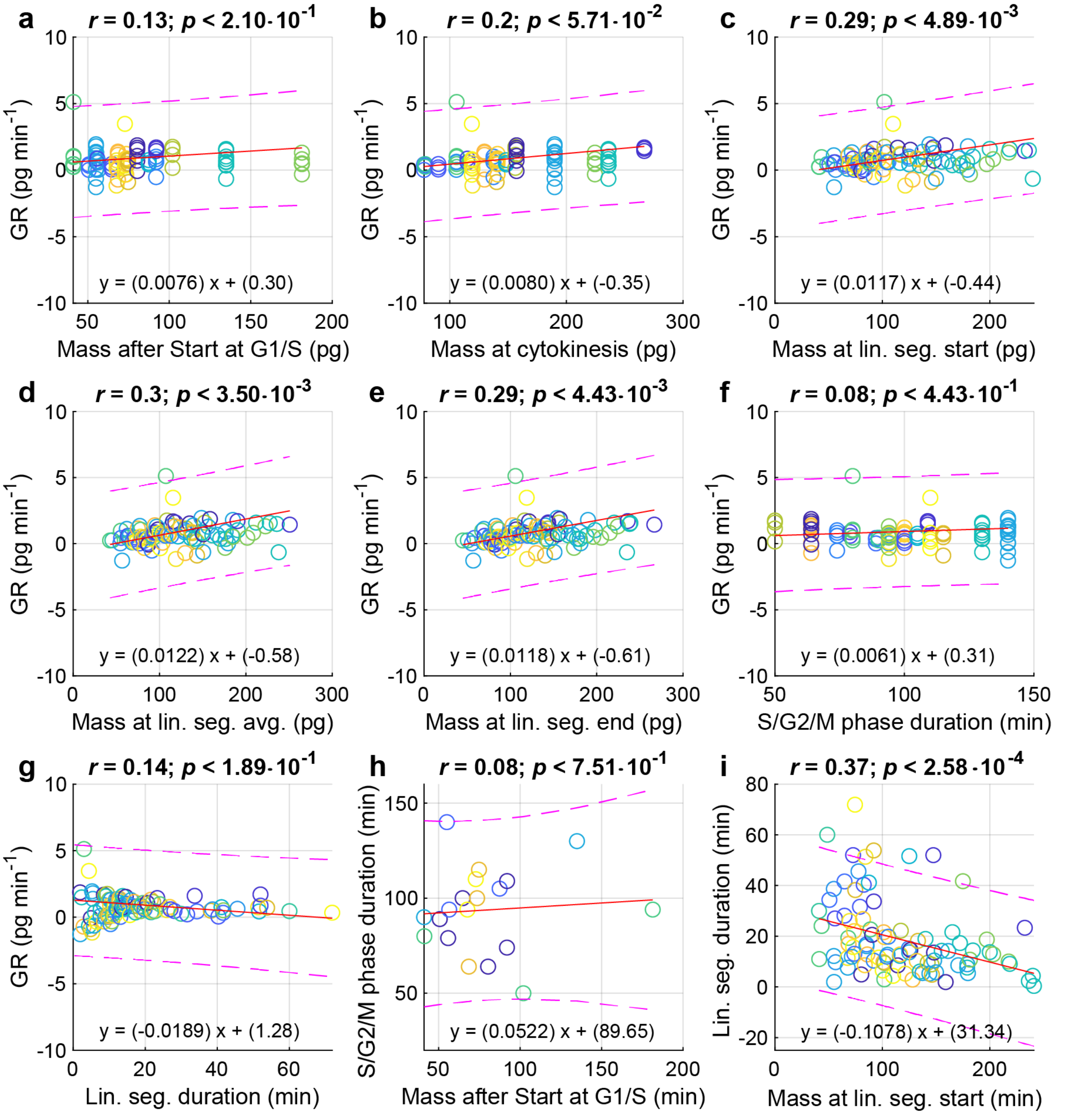

Figure 10. Growth rate analysis with segmented linear model of mass measurements recorded with single yeast cells during the S/G2/M phase. The data originates from (n =19 independent experiments) *S. cerevisiae* cells recorded using the high-resolution continuous mode. Each color group of circles corresponds to a different, individual single yeast cell whereas each circle corresponds to a growth segment. The growth rates (GR) correspond to the slopes of linear segments, which have been identified using a segmented linear regression. Linear fit (red line), 95% prediction interval (dashed lines, magenta) show the general trend. The Pearson correlation (r) and p-value (p) of a two-sided t-test of the linear fit shown above the panels. **a**, GR (colored circles) as a function of the mass after Start[1] at the G1/S transition for the S/G2/M (budding) phase (p-value = 0.2103). **b**, GR (colored circles) as a function of the mass at cytokinesis (cell division) (p-value = 0.057117). **c**, GR (colored circles) as a function of the mass at the onset of each linear

segment (*p*-value = 0.0048946). **d**, GR (colored circles) as a function of the average mass for each linear segment (*p*-value = 0.0034951). **e**, GR (colored circles) as a function of the mass at the end of each linear segment (*p*-value = 0.0044332). **f**, GR (colored circles) as a function of the total duration of the S/G2/M phase (*p*-value = 0.44313). **g**, GR (colored circles) as a function of the duration of each linear segment (*p*-value = 0.18905). **h**, S/G2/M phase duration (colored circles) as a function of the cell mass at the onset of the S/G2/M phase (G1/S transition) for all 19 measured yeast cells (*p*-value = 0.75104). **i**, Segment duration (colored circles) as a function of the cell mass at the onset of each linear segment (*p*-value = 0.00025757).

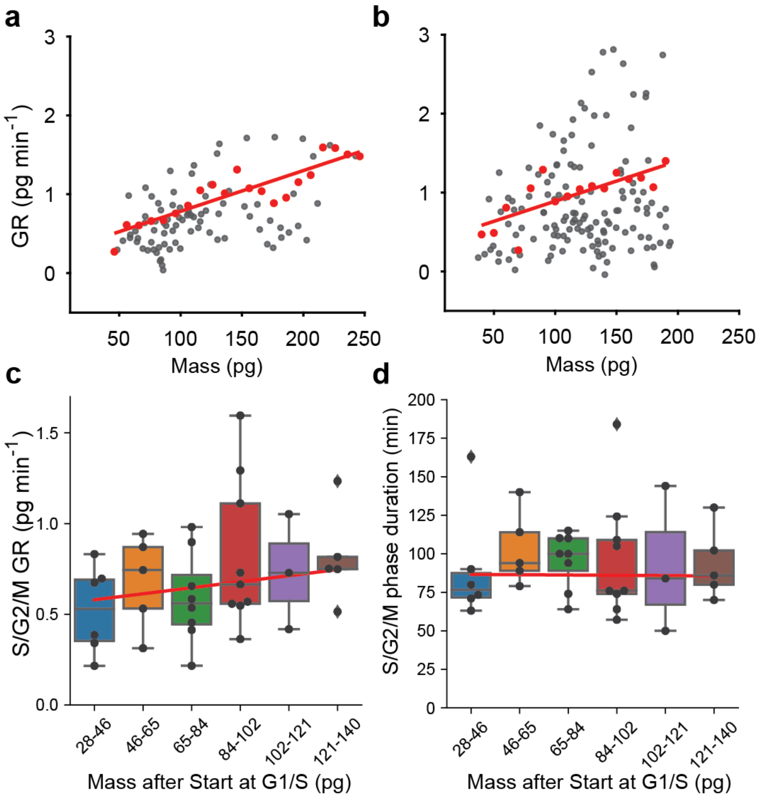

Figure 11. Population analysis shows that on average cells with higher mass at the G1/S transition grow at a higher rate during S/G2/M phase. a, Growth rates (GR as dm/dt) of single yeast cells (n = 19) plotted against their mass m during their S/G2/M phase (individual mass curves in Supplementary Fig. 7). The data was acquired using the continuous mode. Grey circles indicate the data points (fit, r = 0.4, p-value = $2.1655 \cdot 10^{-5}$), red circles the average and the red line is a fit though the averaged data (r = 0.9, p-value = $2.773 \cdot 10^{-8}$. The slope of the fit was found to be 0.0052 min^{-1}. As can be seen, higher masses correlate with higher mass accumulation rates. **b**, As **a** with data (n = 19) being acquired using the sweep mode (individual mass curves are shown in Supplementary Fig. 9). The slope of the fit was found to be 0.0051 min^{-1}. **c**, Mass growth rate of budding yeast in dependence on the start mass as measured at G1/S transition (n = 36 independent experiments from **a** and **b**). The average mass growth rate during S/G2/M was plotted for each cell in a box-plot against the start mass of the cell. For the plot**,** the start masses are binned into six bins of equal size. The center bar of the box plot indicates the median, which was used to fit the red trend line. The bounds of the box indicates the lower $q(0.25)$ and upper quartile $q(0.75)$, in which at least 50% of the data lies. The whiskers extent to show the rest of the distribution, as indicated by the 1.5-fold Inter Quartile Range (limited by minima and maxima). Based on this categorization, the diamonds are considered outliers. The total mass growth rates correlate with the start mass bins with a correlation coefficient of r = 0.6. **d**, S/G2/M phase duration of budding yeast in dependence on the start mass measured at the G1/S transition (n = 36 independent experiments from **a** and **b**). The S/G2/M phase duration was plotted for each cell in a box-plot against the start mass of the cell. Mass after Start[1] at the G1/S transition binned the same way as in **c**. The duration of the S/G2/M phase does not correlate with the start mass. **c**,**d.** Datasets from **a**,**b** have been excluded if the mass exceeded SD = 2 or exceeded a GR SD = 3.

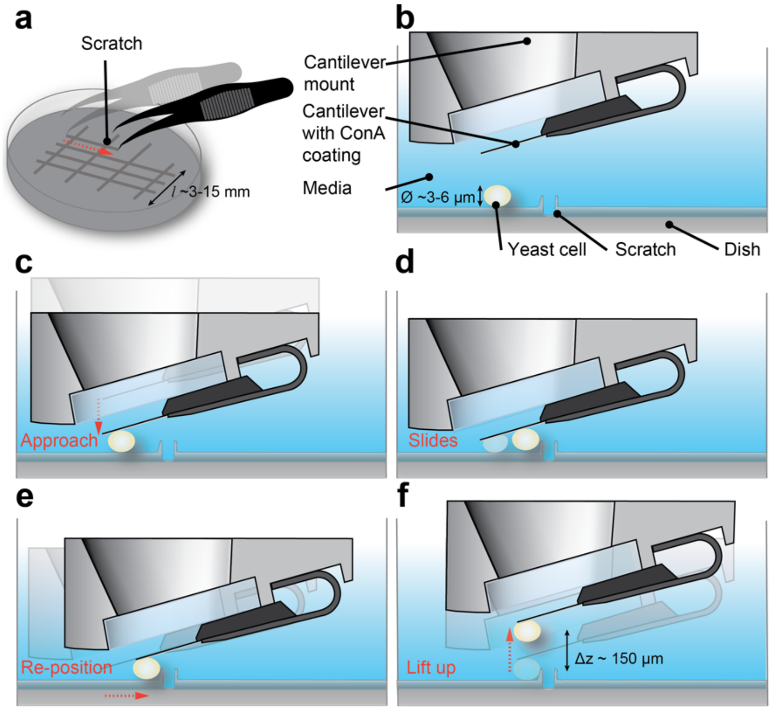

Figure 12. Attachment of single yeast cells to the cantilever. a, Optionally, a pattern as indicated is manually scratched onto the side of a Petri dish using sharp tweezers to facilitate the positioning and attachment. The dish is then filled with a culture of yeast cells in exponential phase and mounted in the environmental chamber of the picobalance where it is kept under culture conditions at 30.0 ± 0.1 °C. **b**, Differential interference contrast (DIC) microcopy is used to localize single yeast cells. **c**, Then, the free end of the concanavalin A (ConA)-coated cantilever of the picobalance is vertically approached onto a yeast cell. In most cases (70%) this procedure attached the cell to the cantilever. **d**, However, sometimes (30%) the slightly tilted (≈ 10°) cantilever upon approaching the yeast cell, pushed the yeast cell away. In such case the yeast cell was pushed towards the edge of a scratch of the Petri dish. **e**, After moving a yeast cell to the edge of the scratch, the free end of the cantilever was vertically pushed onto the cell for attachment. Thereby, the edge of the scratch acts as a barrier preventing the yeast cell to slide away. **f**, To monitor the mass of the yeast cell and to avoid disturbing interactions with the surface of the Petri dish, the cantilever with the adhering yeast cell is retracted from the bottom of the dish by ≈ 100–150 µm. During the mass measurement, the cantilever remains fully immersed in yeast culture medium at cell culture conditions.

Supplementary Note 1

Error estimation of the cell mass using differential interference contrast microscopy

The optical images allow for measuring the diameters of mother and daughter cells during the time course of the mass measurement (Supplementary Fig. 13a). Assuming spherical cells, the analysis confirms that the volume exclusively changes in the budding daughter cell whereas the volume of the mother does not change. This effect has previously been reported by Mitchison and others[2,3]. The measurements further show a good correlation between the total masses monitored by the picobalance and approximated from the cell volume using a density of 1.1 g cm^{-3} for *S. cerevisiae* cells[4,5]. The diameters of mother cell and bud are measured over time using differential interference contrast (DIC) microscopy (Supplementary Fig. 13b). The measurements are used to approximate the volume of the cell and of the bud assuming a spherical geometry. Using the volume, the total mass of cell and bud are calculated taking the density ρ of *S. cerevisiae* of 1.1 g cm^{-3}[4,5]. The first DIC image is taken as a reference and used to calculate the cell mass difference with respect to the other DIC images.

a

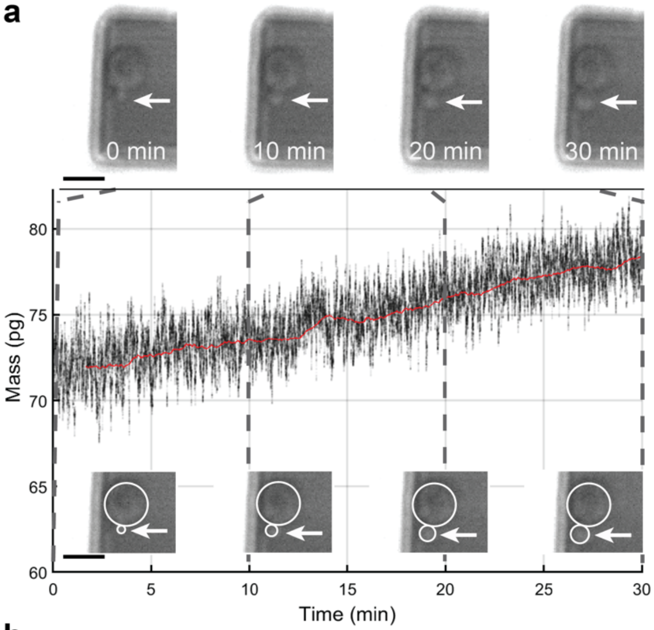

b

Time (min)	0	10	20	30
Mother cell diameter (µm)	5.2 ± 0.6	5.2 ± 0.6	5.2 ± 0.6	5.2 ± 0.6
Daughter cell diameter (µm)	0.9 ± 0.6	1.4 ± 0.6	1.9 ± 0.6	2.2 ± 0.6
Expected mass difference (pg)	—	1.2 ± 2.9	3.5 ± 4.6	5.7 ± 5.9

Figure 13. Cell mass measurements are comparable to the cell mass estimated from optical images. Mass and morphology of a single yeast cell measured at high time and mass resolution in the continuous mode. The mass and microscopy data are taken from Supplementary Fig. 15. Classically the cell mass has

been estimated by measuring the cell diameter from optical images by approximating its volume as a sphere and assuming a constant density parameter. However, mass approximation is prone to even small errors in obtaining the cell diameter. **a**, The cell attached to the microcantilever initiates a bud, which is monitored by both mass and DIC microscopy over the course of 30 min. The black raw data shows the total mass of the cellular process acquired every 10 ms, while the red curve shows the averaged raw data (100 s moving window). The total increase of cell mass is ≈ 6 pg. The diameters of the mother cell and bud are estimated from the DIC images (white circles in bottom images). **b**, The diameter of the mother cell shows no visible change over time, while the bud does. The error in diameter is given by the optical resolution of the measurement (Supplementary Note 1). Assuming spherical shapes for the mother and daughter cells, and a cell density of 1.1 g cm^{-3} for *S. cerevisiae*[4,5], the expected mass difference and propagated errors are estimated. The measured increase of cell mass (≈ 6 pg) correlates well with the expected mass difference associated to the growing bud. The *S. cerevisiae* cell was attached to the cantilever of the picobalance using ConA and the measurement was recorded in yeast culture medium. Scale bars, 5 µm.

The optical resolution, R, when using DIC is given by the following expression:

$$R = \frac{1.22\lambda}{NA_{\text{obj}} + NA_{\text{cond}}} \quad (1)$$

Where λ is the wavelength of the light used, and NA_{obj} and NA_{cond} the numerical aperture of the objective and condenser, respectively. In our setup, the wavelength is 525 nm, and with the numerical aperture of the objective (0.75) and the condenser (0.35) leads to an optical resolution of R = 0.6 µm. We take R as the scale error of the cell diameter E_D in our optical measurement. The errors in the expected total mass difference (Supplementary Fig. 13b) are obtained by error propagation. Given that the diameter of the mother cell does not show any visible change, we calculate the expected total mass difference by subtracting the expected total mass of the bud over time (calculated from the DIC images) from the initial expected total mass of the bud. Let $D_{b,n}$ be the diameter of the bud for the DIC image number n, then assuming a spherical shape for the bud, the volume $V_{b,n}$is:

$$V_{b,n} = \frac{4}{3}\pi\left(\frac{D_{b,n}}{2}\right)^3 \quad (2)$$

and expected mass $M_{b,n}$ can be calculated as using Equation (2):

$$M_{b,n} = \rho \cdot V_{b,n} = \rho\frac{4}{3}\pi\left(\frac{D_{b,n}}{2}\right)^3 \quad (3)$$

Hence, the error $E_{M_{b,n}}$ in the calculated total mass of the bud can be written as:

$$E_{\mathrm{M}_{b,n}} = \left|\frac{\partial M_{b,n}}{\partial \rho}\right| \cdot E_\rho + \left|\frac{\partial M_{b,n}}{\partial V_{b,n}}\right| \cdot E_{V_{b,n}} \qquad (4)$$

Being E_ρ and $E_{V_{b,n}}$ the errors in the cell density and bud volume, respectively. The value for the yeast cell density is obtained from the literature[4,5] and assuming its error is negligible we can write

$$E_{\mathrm{M}_{b,n}} = \left|\frac{\partial M_{b,n}}{\partial V_{b,n}}\right| \cdot E_{V_{b,n}} = \rho \frac{\pi}{2} \left(D_{b,n}\right)^2 \cdot E_D \qquad (5)$$

Therefore, the error for the calculated total mass difference $\left(M_{b,n} - M_{b,1}\right)$ will be $E_{\mathrm{M}_{b,n}} + E_{\mathrm{M}_{b,1}}$, as listed in Supplementary Fig. 13b.

It is important to note that introducing the total mass of the mother cell would not affect the calculated mass differences. However, the errors for estimating the total mass of mother and daughter cell would become much bigger, due to the limitations of optical measurements. To calculate such errors, similar equations as the ones described above can be used, in which the subscript b that stands for the bud and m stands for the mother cell. Doing so, the error of the expected total mass difference between two frames would be $E_{\mathrm{M}_{m,n}} + E_{\mathrm{M}_{b,n}} + E_{\mathrm{M}_{m,1}} + E_{\mathrm{M}_{b,1}}$

Supplementary Note 2

Population level analysis does not reveal segmented linear growth during S/G2/M phase

To compare our high-resolution mass measurements of single cells with bulk data (population level) as observed by others we here study the average growth of many single cells during S/G2/M phase. Specifically, we were interested if we could identify the underlying growth behaviour from averaged single cell mass measurements. Hereto, we first simulated the linear and exponential growth of single cells having different masses and growth rates *in silico* and investigated whether we can extract the underlying growth behaviour from normalized mass and time data of cells progressing through the S/G2/M phase at the population level (Supplementary Fig. 14a, b). The growth rates of the respective models were drawn from a standard normal distribution such that the average mass duplicates over the time of the cell cycle. From this normalized average, the linear model (LM) and exponential model (EM) can clearly be discriminated despite the small differences between linear and exponential growth behaviour (Supplementary Fig. 14a, b bottom row). To normalize our data we normalized the mass of each mass measurement by the average mass of the first 2% of the measurement in the S/G2/M phase and normalized the time of each mass measurement by the duration of the S/G2/M phase (Supplementary Fig. 14c). We then fitted the LM and EM to the normalized data acquired using the continuous mode (Supplementary Fig. 14d) and the sweep mode (Supplementary Fig. 14e). The residuals (fitting deviations), revealed that for the normalized average growth the LM fits the data recorded using the continuous mode (Supplementary Fig. 14d) and sweep mode (Supplementary Fig. 14e) best. However, because the mass data recorded in the sweep mode show lower resolution, their residuals show less prominent differences (Supplementary Fig. 14f). We also observed a short lag in the growth rate for the first 10% of the S/G2/M phase right after G1/S transition (bud emergence) where the residuals for the linear fit deviate from zero (Supplementary Fig. 14c, d), which was consistent with literature[6].

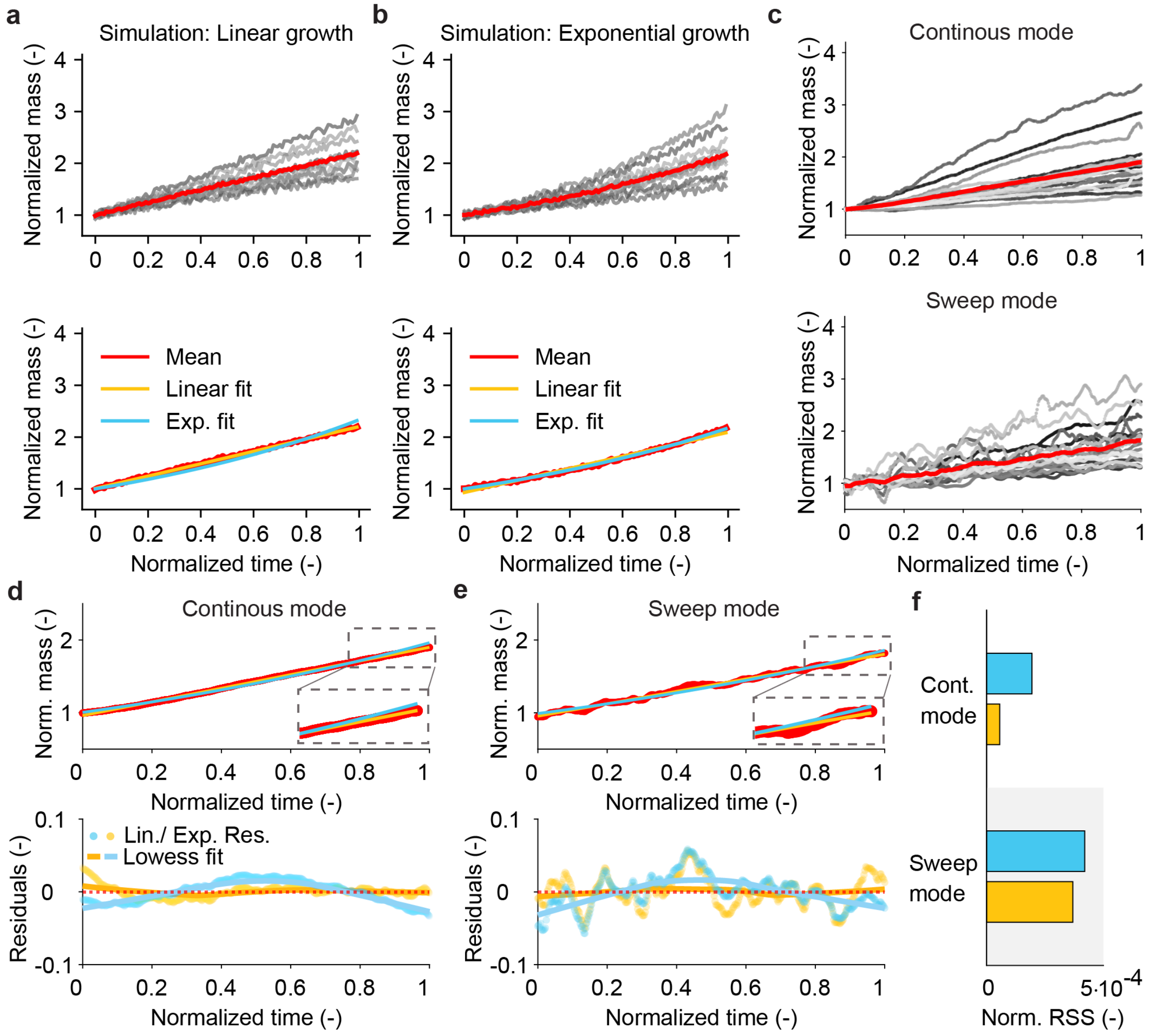

Figure 14. The growth analysis of averaged yeast cells progressing through S/G2/M supports an apparent linear growth behaviour and demonstrates the necessity of high-resolution single-cell techniques. a-e, Normalizing mass and time of each yeast cell progressing through the S/G2/M phase and subsequent averaging to simulate cell growth at the population level. **a**, Top, simulated linear cell growth curves of different cell mass and growth rates were normalized in mass and time (grey) and averaged (red). Bottom, linear model (LM) and exponential model (EM) fitting of the averaged simulated data (red). **b**, Top, simulated exponential cell growth curves of different cell mass and growth rates were normalized in mass and time (grey) and averaged (red). Bottom, LM and EM fitting of the averaged simulated data (red). Please note that the average growth behaviour is not true in general as the sum of exponentials (as used for the averaging) is not an exponential *per se*. However, when the exponentials are similar enough, as it is here due to the restrictions given by the biological background, an exponential can be retrieved after averaging. **c**, The same mass and time normalization shown in **a**, **b** but applied to the average (red) of all individual growth curves (grey) experimentally attained using continuous (top) and sweep (bottom) mode. **d**, Top, EM and LM fits of the averaged (mean) mass data acquired using the continuous mode. Bottom, residuals of linear (yellow) and exponential (cyan) fits. If the fits would capture every change of the growth curve, the residuals would randomly scatter around zero (red dashed), which is not the case for the exponential fit, but is the case for the linear fit for every time point in the normalized time bigger than 0.2. dark yellow and dark cyan lines through the scatter data are locally weighted scatterplot smoothing

(LOWESS) fits showing the trend. **e**, Top, EM and LM fits of the averaged (mean) mass data acquired using the sweep mode. Bottom residuals of linear (yellow) and exponential (cyan) fits. Due to the higher noise of the data acquired using the sweep mode less differences between LM and EM fits are visible. However, the LOWESS fits also show the same trend as for the continuous mode data namely that the LM fits best. **f**, Normalized sum of squared residuals shown in **d** and **f**. In case of the averaged mass data acquired using the continuous mode, the residuals differ by a factor of three.

Supplementary Note 3

Increasing the laser power to increase the mass resolution of the picobalance in the continuous mode

We repeated our experiments with yeast at even higher mass resolution. The frequency noise Δf_N limiting the resolution of the mass measurement is inversely proportional to the oscillation amplitude (Methods). Hence, to enhance the mass resolution of our balance the power of the blue excitation laser can be increased to induce larger oscillation amplitudes. We thus increased the power of the blue excitation laser from ≈ 8 μW to ≈ 28 μW and monitored the mass and morphology of a budding yeast cell (Supplementary Fig. 13 and 15). At this higher laser power mass changes of ≤ 1 pg were observed at 10 ms time resolution. Further, averaging the raw data over time windows of 100 s increased the mass resolution to < 0.2 pg. During the first 30 min of the recording, the mass of mother and daughter cell increased linearly by ≈ 6 pg (Supplementary Fig. 13a). The average growth rate of ≈ 0.2 pg min^{-1} is less than that measured using a lower laser power (≈ 0.6 pg min^{-1}, Fig. 2), but still within the variations reported for *S. cerevisiae* cells[3,7,8] Importantly, the measurement confirmed our observations of small non-uniform deviations from a linear growth.

Monitoring the yeast cell mass at increased high resolution comes with a trade-off. The higher the blue laser power, the more the blue light will be scattered from solution and cantilever, which can harm yeast cell proliferation[9,10]. Additionally, the cantilever partly absorbs the energy of the excitation laser hitting the base, which may also elevate the temperature at its free end to which the yeast cell is attached[10]. Indeed, upon approaching ≈ 60 min the budding process stopped (Supplementary Fig. 15 and Movie 6), indicating that the increased laser power impaired cell growth over an extended period of time. In conclusion, the approach still provides a unique opportunity to study biological processes at very high mass resolution for short intervals of time. For the relatively small cantilevers used in this work, we found that a laser power of ≈ 8 μW had no effect on the morphology and proliferation of yeast cells, even if their mass was monitored for several hours (Supplementary Fig. 2, 3, 4 and Movie 1–3). Nevertheless, for each biological sample, a compromise between mass resolution, duration of the experiment and possible perturbations of the sample must be reached, similarly to routines established for fluorescence microscopy measurements.

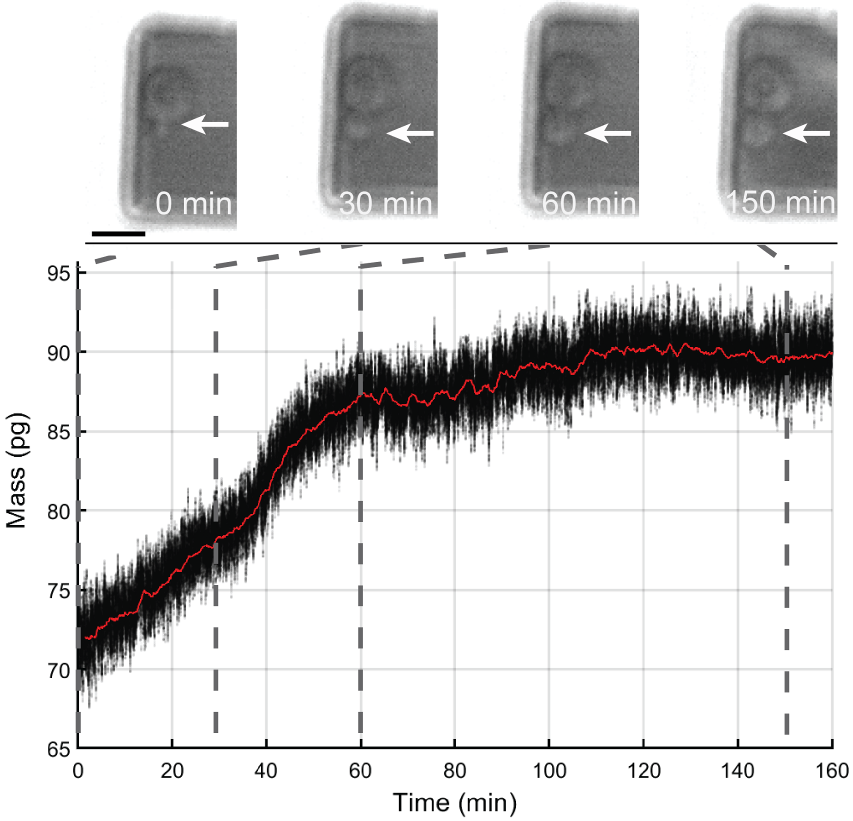

Figure 15. **Increasing the blue laser power can arrest the growth of yeast cells.** We increased the laser power from ≈ 8 to ≈ 24 µW to evaluate the effect of increased blue laser power on yeast growth and obtain higher mass resolution (≤ 1 pg) over long-time mass measurements. The total mass measurements of a budding yeast cell budding were recorded in the continuous mode at a time resolution of 10 ms (black raw data) budding over 160 min. Simultaneously recorded DIC images are shown at the top. The red line, which presents the averaged raw data (100 s moving window), shows a mass resolution of ≤ 0.2 pg. Over the first ≈ 50 min the daughter cell budding from the mother yeast cell increases mass and grows in size (white arrow). After ≈ 60 min, the growth arrests, thus indicating an influence of the increased laser power on cell growth. See also Supplementary Movie 6. Scale bar (black line), 5 µm.

Supplementary References

1. Cross, F. R. Starting the cell cycle: what's the point? *Curr. Opin. Cell Biol.* **7**, 790–797 (1995).
2. Mitchison, J. M. The Growth of Single Cells II. Saccharomyces Cerevisiae. *Exp. Cell Res.* **15**, 214–221 (1958).
3. Ferrezuelo, F. *et al.* The critical size is set at a single-cell level by growth rate to attain homeostasis and adaptation. *Nat. Commun.* **3**, 1–11 (2012).
4. Woldringh, C. L., Huls, P. G. & Vischer, N. O. E. Volume Growth of Daughter and Parent Cells during the Cell-Cycle of Saccharomyces-Cerevisiae a/Alpha as Determined by Image Cytometry. *J. Bacteriol.* **175**, 3174–3181 (1993).
5. Baldwin, W. W. & Kubitschek, H. E. Buoyant density variation during the cell cycle of Saccharomyces cerevisiae. *J. Bacteriol.* **158**, 701–704 (1984).
6. Goranov, A. I. *et al.* The rate of cell growth is governed by cell cycle stage. *Genes Dev.* **23**, 1408–1422 (2009).
7. Lord, P. G. & Wheals, A. E. Variability in Individual Cell-Cycles of Saccharomyces-Cerevisiae. *J. Cell Sci.* **50**, 361–376 (1981).
8. Li, S., Giardina, D. M. & Siegal, M. L. Control of nongenetic heterogeneity in growth rate and stress tolerance of Saccharomyces cerevisiae by cyclic AMP-regulated transcription factors. *Plos Genet.* **14**, 1–42 (2018).
9. Schmidt, G. W., Cuny, A. P. & Rudolf, F. Preventing photomorbidity in long-term multi-color fluorescence imaging of saccharomyces cerevisiae and S. Pombe. *G3 Genes, Genomes, Genet.* **10**, 4373–4385 (2020).
10. Tinevez, J. Y. *et al.* A Quantitative Method for Measuring Phototoxicity of a Live Cell Imaging Microscope. *Imaging Spectrosc. Anal. Living Cells Imaging Live Cells Heal. Dis.* **506**, 291–309 (2012).
