# Supplementary material for: High-resolution mass measurements of single budding yeast reveal linear growth segments
Source: Nat Commun. 2022 Jun 22;13:3483. doi: 10.1038/s41467-022-30781-y (PMC9217925; doi:10.1038/s41467-022-30781-y)
Supplement: Supplementary file 2 — Reporting Summary [file 41467_2022_30781_MOESM2_ESM.pdf]

nature research

Corresponding author(s): Daniel J. Müller

Last updated by author(s): May 26, 2022

Reporting Summary

Nature Research wishes to improve the reproducibility of the work that we publish. This form provides structure for consistency and transparency in reporting. For further information on Nature Research policies, see Authors & Referees and the Editorial Policy Checklist .

Statistics

For all statistical analyses, confirm that the following items are present in the figure legend, table legend, main text, or Methods section.

n/a	Confirmed	
☐	☒	The exact sample size (*n*) for each experimental group/condition, given as a discrete number and unit of measurement
☐	☒	A statement on whether measurements were taken from distinct samples or whether the same sample was measured repeatedly
☐	☒	The statistical test(s) used AND whether they are one- or two-sided<br>*Only common tests should be described solely by name; describe more complex techniques in the Methods section.*
☒	☐	A description of all covariates tested
☐	☒	A description of any assumptions or corrections, such as tests of normality and adjustment for multiple comparisons
☐	☒	A full description of the statistical parameters including central tendency (e.g. means) or other basic estimates (e.g. regression coefficient) AND variation (e.g. standard deviation) or associated estimates of uncertainty (e.g. confidence intervals)
☐	☒	For null hypothesis testing, the test statistic (e.g. *F*, *t*, *r*) with confidence intervals, effect sizes, degrees of freedom and *P* value noted<br>*Give P values as exact values whenever suitable.*
☒	☐	For Bayesian analysis, information on the choice of priors and Markov chain Monte Carlo settings
☒	☐	For hierarchical and complex designs, identification of the appropriate level for tests and full reporting of outcomes
☐	☒	Estimates of effect sizes (e.g. Cohen's *d*, Pearson's *r*), indicating how they were calculated

Our web collection on statistics for biologists contains articles on many of the points above.

Software and code

Policy information about availability of computer code

Data collection	The mass data has been acquired with Nanosurf's software (version 2019, Nanosurf AG, Liestal, Switzerland) and custom LabVIEW 2019 (version 2019, National Instruments) scripts. The image data has been acquired with the Nikon NIS Elements (version 5.21, Nikon Europe B.V., Egg, Switzerland) software.
Data analysis	The mass data has been analyzed with pyIMD (version 0.1.3) available from (https://gitlab.com/csb.ethz/pyIMD). The data has been processed and analyzed with custom MATLAB (version R2021a, MathWorks, Natick, MA, USA) scripts or with Python (version 3.7.7) using Jupyter notebooks to generate the figures. The scripts are available together with the data (https://www.doi.org/10.3929/ethz-b-000547242).

For manuscripts utilizing custom algorithms or software that are central to the research but not yet described in published literature, software must be made available to editors/reviewers. We strongly encourage code deposition in a community repository (e.g. GitHub). See the Nature Research guidelines for submitting code & software for further information.

Data

Policy information about availability of data

All manuscripts must include a data availability statement. This statement should provide the following information, where applicable:

- Accession codes, unique identifiers, or web links for publicly available datasets
- A list of figures that have associated raw data
- A description of any restrictions on data availability

All data generated and used in this study in its raw and processed form are freely available and have been deposited in the ETH Research Collection under the accession code DOI https://www.doi.org/10.3929/ethz-b-000547242.

Field-specific reporting

Please select the one below that is the best fit for your research. If you are not sure, read the appropriate sections before making your selection.

- [x] Life sciences
- [ ] Behavioural & social sciences
- [ ] Ecological, evolutionary & environmental sciences

For a reference copy of the document with all sections, see nature.com/documents/nr-reporting-summary-flat.pdf

Life sciences study design

All studies must disclose on these points even when the disclosure is negative.

Sample size

No sample size calculations were performed. The sample size (n) of each experiment is provided in the corresponding figure captions of the main manuscript and supplementary information. In brief, this study presents the cell mass of n=38 independently measured yeast cells over at least one cell cycle with accompanying microscopy data acquired on different days. Sample sizes were chosen to support meaningful conclusions between two presented measurement modes (High res. continuous mode versus Sweep mode with each n=19).

Data exclusions

The exclusion criterion were predetermined and defined by the measuring system. We focused mainly on the S/G2/M phase of the yeast cell cycle which is the phase when a bud emerges from a single budding yeast cell until the cell division. This phase was determined with the help of microscopy images and fluorescently labeled markers inside the cells as described in the Methods section in detail. The short G1 phase we did not analyze further due to the small sample size (n=10). However, the yeast cells typically grew for at least two cell cycles (generations) and many hours on the cantilever except for the experiments performed with high laser power which stopped cell growth after roughly 30 mins intentionally. Please note that the culturing conditions were only maintained for the data presented. In some cases the system recorded the data longer (i.e over night) to not loose any data. Even-dough the microscopy images show viable, dividing cells on the cantilever for such later phases and time-points, this data does not necessarily correspond to the mass of a single cell anymore and proper attachment of such cell colonies to the cantilever is not guaranteed as discussed in the manuscript and supplementary information.

Replication

Mass experiments were performed for 38 independent replicates of genetically identical yeast cells, each yielding consistent results measured with two independent measuring modes (continous and sweep mode). Additionally independent control measurements (no cell attached to the cantilever) have been performed multiple times successfully reproducing the reported stability over several hours at high time resolution of 10 ms in the continous mode.

Randomization

Not relevant to this study. The study does not involve participant groups. Each experiment is based on a randomly selected, newborn cell from the same genetic background under identical culturing conditions performed by two independent authors as described in the Methods section in detail.

Blinding

Predetermined automated analysis without human interference except for the cell cycle phase determination. The cell cycle phase determination has been performed by three authors independently with the help of microscopy images and fluorescently labeled markers inside the cells indicating their intrinsic state as described in the Methods section in detail. The experiment group allocation was predetermined by the measurement mode used (continous versus sweep mode versus control). The experiments have been performed by two independent authors both using each mode on randomly selected cells. This study does not involve participant groups. Therefore, blinding was not relevant to the study.

Reporting for specific materials, systems and methods

We require information from authors about some types of materials, experimental systems and methods used in many studies. Here, indicate whether each material, system or method listed is relevant to your study. If you are not sure if a list item applies to your research, read the appropriate section before selecting a response.

Materials & experimental systems

n/a	Involved in the study	
x		Antibodies
	x	Eukaryotic cell lines
x		Palaeontology
x		Animals and other organisms
x		Human research participants
x		Clinical data

Methods

n/a	Involved in the study	
x		ChIP-seq
x		Flow cytometry
x		MRI-based neuroimaging

Eukaryotic cell lines

Policy information about cell lines

Cell line source(s)	All cell lines are derived from prototrophic FRY2032. This is a derivative of the diploid FRY2023 that originates from crossing FRY1398 MAT a, FY4 (BY4700) and FRY1400, MAT alpha, FY4 (BY4707) which come from the following reference: Gnugge, R., Liphardt, T. & Rudolf, F. A shuttle vector series for precise genetic engineering of Saccharomyces cerevisiae. Yeast 33, 83-98, doi:10.1002/yea.3144 (2016).
Authentication	Cell lines were authenticated by microscopy and loss of of auxotrophies confirmed by growth on minimal media.
Mycoplasma contamination	The cell lines were not tested for mycoplasma contamination.
Commonly misidentified lines (See ICLAC register)	No commonly misidentified cell lines were used in the study.
