# Supplementary material for: High-resolution mass measurements of single budding yeast reveal linear growth segments
Source: Nat Commun. 2022 Jun 22;13:3483. doi: 10.1038/s41467-022-30781-y (PMC9217925; doi:10.1038/s41467-022-30781-y)
Supplement: Supplementary file 3 — Description of Additional Supplementary Files [file 41467_2022_30781_MOESM3_ESM.pdf]

Title: Supplementary Movie 1.
Description: Monitoring the mass and morphology of a single *S. cerevisiae* cell adhering to a microcantilever. The mass measurements were recorded using the high-resolution continuous mode. The movie shows the morphology (differential interference contrast (DIC) images) and mass measurement of a yeast cell described in Fig. 2a.

Title: Supplementary Movie 2.
Description: Monitoring the mass and morphology of a single *S. cerevisiae* cell over multiple cell cycles (generations) on the microcantilever during mass measurements. The mass measurements were recorded using the high-resolution continuous mode. The movie shows the morphology (DIC images) and mass measurement of a yeast cell described in Supplementary Fig. 2.

Title: Supplementary Movie 3.
Description: Single *S. cerevisiae* cells increase mass in segments at different rates over the cell cycle for consecutive budding events. The mass measurements were recorded using the high-resolution continuous mode. The movie shows the morphology (DIC images) and mass measurement of a budding yeast cell described in Supplementary Fig. 3.

Title: Supplementary Movie 4.
Description: Monitoring the mass, morphology, and cell cycle phases of a single *S. cerevisiae* cell using fluorescence microscopy. The movie shows the morphology, cell cycle phases, and mass measurement of the yeast cell described in Fig. 3a. The fluorescence Whi5 signal (magenta, mKOκ (1x)) in the nucleus marks the G1 phase and the fluorescence Myo1 signal (cyan, mKate2 (3x)) marks the S/G2/M phase of the cell cycle. The composite movie shows the superimposition of DIC images and both fluorescence signals.

Title: Supplementary Movie 5.
Description: Mass dynamics and time-lapse of a single *S. cerevisiae* cell recorded in the sweep mode. The movie shows the morphology (DIC images) and mass measurement of the yeast cell described in Supplementary Fig. 5.

Title: Supplementary Movie 6.
Description: Evaluating the effect of increasing the blue laser power over longtime mass measurements. The movie shows the morphology (DIC images) and mass measurement of the yeast cell described in Supplementary Fig. 15.
